# Books Review

**Published:** 1870-02

**Authors:** 


### Books Review.

*A Practical Treaties on the Diseases of Children,* By ALFRED VOGEL, M. D. Translated and Edited by H. RAPHAEL, M. D. (*From the Fourth German Edition.*) D, APPLETON & COMPANY: NEW YORK, 1870.

This work upon the diseases of Children, commends itself to our approval, though we have not yet been able to examine it as minutely as its importance demands. There are many points of merit of which we are ready to speak, the first of which is: it will be found to contain a more or less detailed account of every morbid condition known to affect our race in infancy and childhood. The second point of merit will be found in the careful consideration bestowed upon the causes of these various accidents and ills. Another marked attraction of the work is, the completeness of the descriptions of disease. A work having these merits, will necessarily be believed to also contain correct and complete therapeutical conclusions.

Our attention is attracted to one paragraph as we turn its pages, in the etiology of hereditary syphilis, which strikes us with some surprise. Page 590, our author says: "In the great majority of cases, hereditary syphilis decends from the father not the mother. If the mother is affected with secondary syphilis, the pregnancy will hardly ever go on to natural conclusion; an abortion, or, at least, a premature delivery will take place. This in fact, happens also, although less frequently, in secondary syphilis of the father; the pregnancy here, usually terminates normally, but the child comes into the world either with pemphigus syphilitica, or manifest signs of hereditary syphilis during the first six months ot life."

We had thought that it had never yet been fully shown, that the child inherited syphilis of the father, unless through the mother secondarily. Many authors still teach the doctrine, that syphilis in the mother is communicated to the child, while this disease in the father, is rarely, probably never thus extended. However, upon these points, differences of opinion must be expected.

The work is illustrated by six lithographic plates, which add greatly to its value. It appears to us from the examination we have been able to make, that perhaps the whole subject of the diseases of children is more fully and more perfectly embodied in this book than in almost any other.

---

*Plastic Operations. A Paper read before the New York Academy of Medicine.* By PROF. FRANK H. HAMILTON.

In this paper is described a large number of operations of Plastic Surgery, and the results, so far as known, of the operations. Many of the cases occurred in Prof. Hamilton's practice while resident in this city. In one case, that of Dr. Ward's of Silver Creek, operated upon for epithelioma, it should be added that the disease has returned, but still makes very slow progress. The cases are instructive and the modes of operation are ingenious.

*Sawyer's Obstetric Aphorisms.* By JOSEPH GRIFFITHS SWAYNE, M. D., & EDWARD R. HUTCHINGS, M. D. HENRY C. LEA, 1870.

This book is "all about Obstetrics" in few pages, and is eminently adapted to the end in view, being designed for students, and those commencing the practice of obstetrics. It is certainly well arranged for presenting the main and essential facts in this department, and would serve as an excellent guide, until more complete instructions could be obtained.

---

*Transactions of the American Ophthalmological Society and Otological Society.*

The Sixth Annual Meeting of the Ophthalmological Society was held July 21st, 1869, at Newport, R. I. Drs. B. Joy Jeffries, of Boston; H. Knapp, of New York; D. B. St. John Roosa, of New York; John Green of St. Louis, Mo ; Edward G. Loring. of New York; Henry D. Noyes, of New York; E. Dyer, Philadelphia; C. R. Agnew, of New York, and G. Hay, of Boston. presented valuable papers upon the various subjects of Ophthalmology.

The Otological Society held, also, its Second Annual Meeting at Providence. R. I., July 20th, and the reports of these societies are published in connection with each other. Many interesting subjects connected with the objects of the society were presented and discussed by the members. The members seem deeply interested in promoting, not only the interests of these societies but the advancement of their favorite departments of medicine.

---

*On the Wasting Diseases of Infants and Children.* By EUSTACE SMITH, M. D., London. Philadelphia, HENRY C. LEA, 1870.

This volume, of about two hundred pages, is devoted to a consideration of the various defects of nutrition grouped and vaguely expressed by the terms, "Marasmus," "Tabies," "Atrophy," &c. The aim has been to define, as far as possible, and describe all the various conditions upon which these "wasting" diseases depend, and present them to the profession in definite and available forms. The work is based upon ample personal observation, and every chapter shows labor and thought, turned, certainly, in the right direction. We regard it as a valuable contribution to the literature of the diseases of children. It will be found a practical guide of great value in the treatment, especially, of these forms of diseases.

---

*Diseases and Injuries of the Eye; their Medical and Surgical Treatment.* By GEORGE LAWSON, F. R. C. S. Philadelphia, LINDSAY & BLAKISTON, 1869.

This is a manual comprising the work of the author formerly published upon injuries of the Eye, together with a condensed treatise upon the diseases of this organ, and the best methods of treatment. In its present form it is one of our most attractive and complete manuals upon these subjects, and commends itself to the attention of students and practitioners as constituting a sufficient practical guide in most instances. If students and practitioners will acquaint themselves with the teachings of this book, which the author modestly styles a "Manual" they can no longer be regarded as ignorant of the science and art of Ophthalmology, or as unprepared to treat diseases and injuries of the eye. Hand-books of medicine and surgery appear too much like "offering the rewards of diligence without suffering its fatigues," but it cannot be denied that this and other similar condensed works upon this subject have contributed much to general knowledge of the diseases and injuries of the eye, a branch of surgery which has been almost wholly neglected by the general practitioner. We, therefore, most earnestly recommend it to the attention of the profession, and trust that a class of diseases so important to the public and the profession, will receive the study they so much require.

